# Decomposing social groups differential in stunting among children under five in India using nationally representative sample data

**DOI:** 10.1038/s41598-024-78796-3

**Published:** 2024-11-08

**Authors:** Mriganka Dolui, Sanjit Sarkar

**Affiliations:** Department of Geography, Central University of Karnataka, Karnataka, 585311 India

**Keywords:** Stunting, Malnutrition, Inequality, Fairlie decomposition, And India, Malnutrition, Public health, Health care

## Abstract

Stunting among children is a reflection of the chronic malnutrition caused by a complex set of behavioural, demographic, and socioeconomic factors. This long-term detrimental exposure to chronic malnutrition is disproportionately higher among social and economically deprived groups, leading to significant differentials in the prevalence of stunting across various social strata. Therefore, this study investigates the inequality of social groups in terms of the prevalence of stunting across Schedule Caste (SC)-Scheduled Tribe (ST) and non-SC-ST. The study used 1,93,886 children’s data aged 0–59 months from the recent round of the National Family Health Survey. Descriptive statistics, multivariable logistic regression, F-test, t-test and chi-squared (χ²) test were applied to understand the prevalence, determinants, and associations, respectively. The Fairlie decomposition model was applied to quantify the factors contributing to the inequality of stunting across social groups. The results revealed that the prevalence of stunting was higher among SC-ST (39.60%) children compared to non-SC-ST (33.27%). In addition, children aged 15–30 months (AOR: 1.895, 95% CI: 1.843–1.949), and male (AOR: 1.074, 95% CI: 1.053–1.095), mothers had lower BMI (AOR: 1.543, 95% CI: 1.492–1.595), mothers who had no education (AOR: 1.595, 95% CI: 11.532–1.662), belongs to poorest wealth index (AOR: 1.857, 95% CI: 1.766–1.952), and the children belong to the household with unhygienic satiation practices (AOR: 1.097, 95% CI: 1.070–1.123) were more likely to be stunted. The decomposition results revealed that the variables included in the study could explain 68.9% of the stunting inequality between SC-ST and non-SC-ST groups. The household’s wealth index is found to be a leading factor, which contributed nearly 41.3% of total stunting inequality exists between these two groups, followed by mothers’ education (12.86%) and mothers’ BMI (11.02%), sanitation facilities (4.26%), children’s birth order (3.32%) and mother’s type of delivery (1.49%). These findings emphasize the importance of targeted interventions. Prioritizing policies that address household economic enhancement, women’s education and empowerment can be instrumental in reducing social group inequality and lowering the overall prevalence of stunting. Ensuring access to improved hygienic sanitation facilities in the household is equally important for achieving better health outcomes for the children.

**Supplementary Information:**

The online version contains supplementary material available at 10.1038/s41598-024-78796-3.

## Introduction

Stunting is the adverse effect of chronic malnutrition due to long-term inadequate consumption of nutrition and coexisting illness among children^1^. It has long-term detrimental health effects, such as congenital issues, slower cognitive development, and reduced educational achievement^2,3^. Stunting decreases children’s height permanently and can impact future generations, leading to lower income and earnings in adulthood due to lifelong short stature^4^. The issue of stunting is a complex phenomenon resulting from the dynamic interaction of insufficient dietary consumption, food insecurity, household economy, education, occupation and environmental issues^5^. Stunting causes more than one million child deaths each year around the world. Between 2012 and 2023, the prevalence of stunting in Asia decreased by 22.3% from 28.2% ^6^. In 2018, developing countries such as India experienced the highest rate of malnutrition in the world, accounting for 47 million stunted children^7^.

According to the National Family Health Survey (NFHS), the prevalence of stunting in India reduced from 38 to 36% between 2015 and 16 and 2019–2021 ^8^. Even though NFHS-5 indicated that stunting had risen substantially among children aged 6 to 23 months. Furthermore, children in the lowest wealth quantile had a higher stunting prevalence of 46%. Nonetheless, the backward social groups such as Schedule Caste (SC) and Scheduled Tribe (ST) are more vulnerable to stunting, with rates of 39.2% and 40.9%, respectively, compared to general castes and Other Backward Classes (OBC)^8^. Few studies conducted in India revealed that social and caste groups have a substantial impact on influencing health outcomes^9,10^. Stunting is not only a health concern but a manifestation of the pervasive social hierarchy and discrimination experienced by vulnerable populations^11^. However, studies revealed that children from the lowest wealth quintiles and those SC-ST groups are much more prone to stunting, highlighting the convergence of caste, poverty, and health inequities in India^12,13^.

The enduring nature of social group inequalities in child nutrition underscores the necessity of examining stunting through the perspective of caste-based inequities. Stunting among social and caste groups highlights the convergence of structural inequities, historical marginalization, and systemic barriers to access to vital services, including healthcare, nutrition, and education of mothers^14^. Studies have shown that children from lower wealth quintiles and marginalized socioeconomic groups, especially ST-STs, are more significantly impacted by stunting than non-SC-STs^11,12^. These groups often reside in rural areas, are geographically isolated, hold distinctive traits, shyness of contact with other communities and backwardness where infrastructure and social safety nets are deficient, further exacerbating their vulnerability to stunting^11^. It is well-known that maternal chronic energy deficiency is one of the leading risk factors for children’s growth retardation in India^15^. These populations encounter structural obstacles that intensify nutritional inadequacies and restrict prospects for upward mobility^14^.

Additionally, child-related characteristics such as child age, birth order, child’s sex, antenatal care visit (ANC), place of residence and geographical variation have an impact on the increased prevalence of stunting^16–21^. However, most of the prior research had focused on the prevalence and association of stunting. A few limited studies from India have revealed that stunting is severely concentrated among socially disadvantaged groups, particularly in SC and STs, then in other social groups^18,22^. At the same time, caste-based (SC-ST and non-SC-ST) inequality in nutritional outcomes may have a potential footprint in pointing out the factors that trigger social discrimination. Therefore, this study aims to investigate the inequality in stunting and the factors contributing to the gap among SC-ST and non-SC-ST social groups in India.

## Methods

### Data source

This study uses the National Family Health Survey fifth-round (NFHS-5) (2019–2021) data. The NFHS provides nationally representative data that collect information on a wide range of demographic, socioeconomic, maternal and child health, nutrition, reproductive, and family planning issues across the districts and states/UT in India at both household and individual levels^8^. The survey followed a two-stage stratified sampling procedure conducted by the International Institute of Population Sciences (IIPS) under the Ministry of Health and Family Welfare (MoHFW) of the Government of India^8^.

**Fig. 1:**
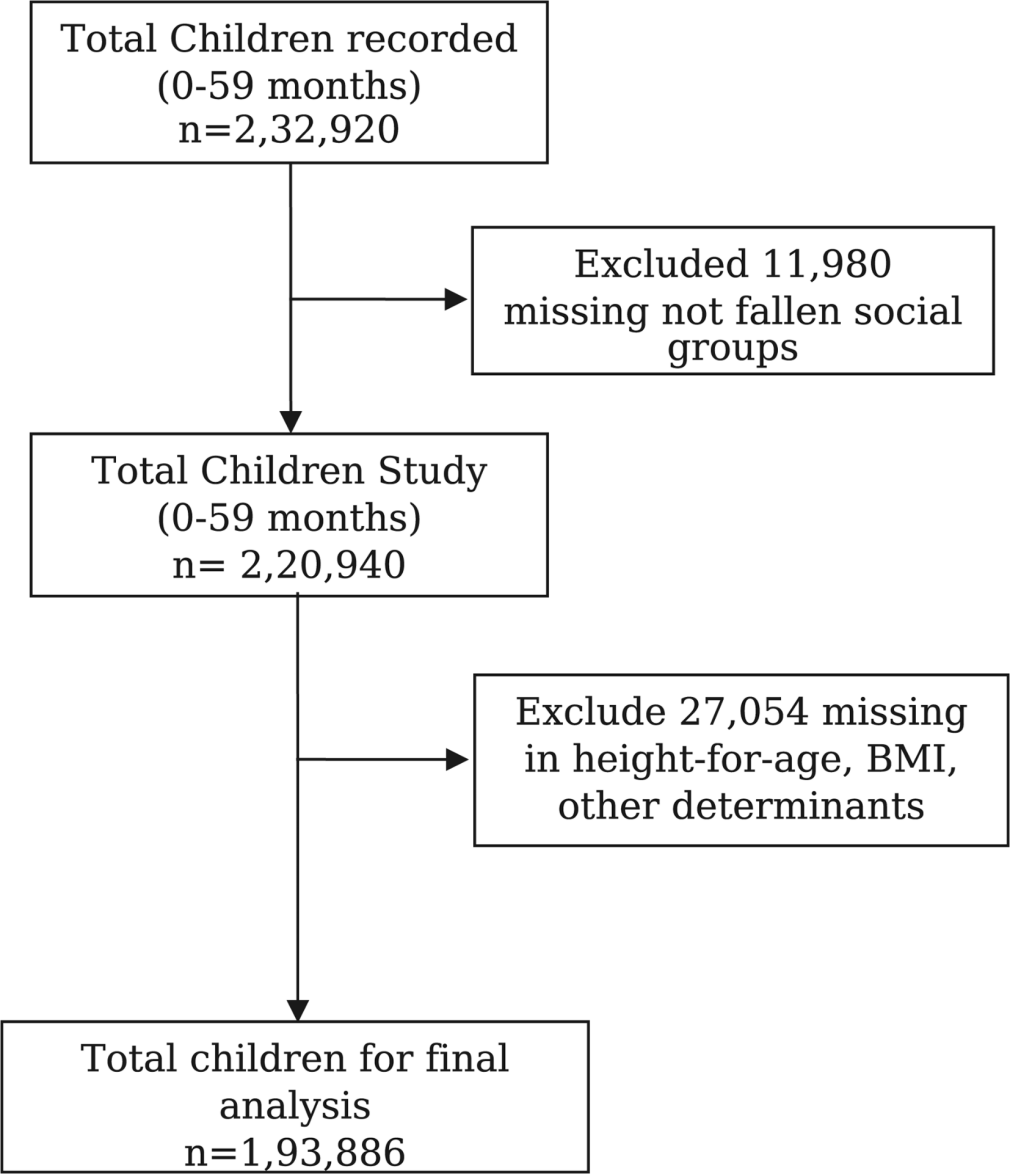
Selection of sample size for the study.

For this study, the sample was restricted to children aged 0–59 months. The NFHS-5 recorded 2,32,920 children’s information for children aged 0–59 months, including anthropometric (height and weight) information. Among them, 11,980 children were excluded from our analysis due to missing in social category. Subsequently, from the remaining 2,20,940 children aged 0–59 months, 27,054 missing in were excluded due to missing information on height-for-age, BMI, and other determinants. Hence, a final sample of 1,93,886 children aged 0–59 months was considered for the study. For the social group, we have classified the samples into two categories: SC-ST (*n* = 83,909) and Non-SC-ST (general castes and OBC) (*n* = 1,09,977). A detailed description of sample selection is presented in Fig. 1.

## Outcome variable

The outcome variable of our study was stunting (height-for-age). During the NFHS-5 survey, the interviewer measured children’s height and weight through an Anthropometric measurement tool. Further, children’s height/length and age data were used to calculate stunting (height-for-age)^8^. As per World Health Organization (WHO) standards guidelines, children whose height-for-age Z-score (HAZ) value was below minus two standard deviations (< -2SD) were considered as stunting (HAZ < -2)^23,24^. Therefore, for preliminary analysis, we used height-for-age as a continuous variable, and further, the outcome variable was converted into a dichotomous for the purpose of analysis i.e., stunted if HAZ < -2 SD and otherwise non-stunted.

## Explanatory variables

The present study included the relevant demographic and socioeconomic variables based on existing literature on stunting^1,4,16,18,25–29^. The selected variables are potentially efficient in explaining the associations and inequality of stunting. The explanatory variables include the caste of the children (SC-ST and Non-SC-ST), age of the children (< 15, 15–30, 31–45, and 46–59 months), gender of the children (male and female), birth order (1, 2, and 3+), mother’s age at birth (< 20, 20–30, and > 30 years), mother’s delivery (normal and caesarean), mother’s BMI (underweight, normal, and overweight), religion (Hindu, Muslim, and others religion), education of mothers (no education, primary, secondary, and higher), working status (not working and working), wealth index (poorest, poorer, middle, richer, and richest), place of residence (urban and rural), geographical region (north, central, east, northeast, west, and south), drinking behaviour (alcohol) of mother’s (yes and no), smoking behaviour (yes and no), use of cooking fuel (clean fuel and polluting fuel), status of sanitation (safe and unsafe) and drinking water facility (safe and unsafe) as covariable.

The wealth index variable, available in the unit-level data, was computed using wealth scores derived from principal component analysis (PCA), considering indicators such as consumer goods ownership and housing features by NFHS and a detailed methodology mentioned elsewhere^8^. Following this, households were divided into five equal groups, known as quintiles or wealth quintiles. The wealth quintiles were further categorized into 20% each, including the poorest, poorer, middle, richer, and richest^8^. Whereas the geographical region was categorised based on the direction of the provided states^30^. Furthermore, smoking behaviour was defined as those who consume any of the substance, including cigarettes/pipes full of tobacco/ smoking cigars, cheroots or cigarillos/ water pipe or hookah/ smokes or use gutkha or paan masala with tobacco/ smokes or uses khaini/smokes termed as yes, else no^31^. Similarly, for the use of cooking fuel, those who use electricity, Liquified Petroleum Gas (LPG) and biogas for cooking are defined as clean cooking fuel, and those who use kerosene, coal-lignite, charcoal, wood, straw/shrubs/grass, agricultural crops, and animal dung termed as polluting cooking fuel^32^. Then, for sanitation status, safe sanitation practices are defined as those who use flush-to-piped sewer systems, flush-to-septic tanks, flush-to-pit latrines, ventilated improved pit latrines (VIP), pit latrines with slab, and those using other than these methods termed as unsafe sanitation practices^33^. Likewise, for drinking water facilities, safe drinking water was defined as those who drink water from piped water, piped into dwelling, piped to yard/plot, piped to neighbour, public tap/standpipe, tube well or borehole, protected well, protected spring, tanker truck, cart with small tank, bottled water, community or plant, and drink from any other sources termed as unsafe drinking water^33^.

### Statistical analysis

We utilized a combination of descriptive statistics and bivariate and multivariable analysis to understand the status of stunting and inequality among SC-ST and non-SC-ST children in India. First, we calculated the prevalence of stunting (HAZ < -2) and mean Z-score HAZ of among SC-ST and non-SC-ST children. In addition, we have evaluated the significance of the association between each explanatory variable and stunting using the chi-square (χ²) test (for dichotomous), F-test and t-test (for continuous). Next, we employed a multivariable logistic regression model at a 95% significance level to identify the factors associated with stunting. Subsequently, we measured the social group inequality in stunting between SC-ST and Non-SC-ST and their contribution to stunting using the “Fairlie” decomposition model.

First, the multivariable logistic regression models of stunting of SC-ST and non-SC-ST children were analyzed by the following equation:

$$\:\text{ln}\left(\frac{{p}_{i}}{1-{p}_{i}}\right)=\alpha\:+{\chi\:}_{1}{\beta\:}_{1}+{\chi\:}_{2}{\beta\:}_{2}+{\chi\:}_{k}{\beta\:}_{k}+\: \text{\euro}$$

Where$\:,\:\:\text{ln}\left(\frac{{p}_{i}}{1-{p}_{i}}\right)$ is the odds in which *p*_*i*_ is the probability of ‘i’ individual experiencing the outcome events, $\:\alpha\:$ is the constant, χ_i_ is the vector of the predictor variables, β_i_ is the vector of regression coefficients, and $\: \text{\euro}$ is the unexplained part or error term. After that, the differences in stunting between SC-ST and Non-SC-ST children were decomposed as:

$$\:{\overline{\text{Y}}}^{\text{a}}-\:{\overline{\text{Y}}}^{\text{b}}=\left[\sum\:_{i-1}^{{N}^{a}}\frac{F\left({X}_{i}^{a}{\beta\:}^{a}\right)}{{N}^{a}}-\sum\:_{i-1}^{{N}^{b}}\frac{F\left({X}_{i}^{b}{\beta\:}^{a}\right)}{{N}^{b}}\right]+\left[\sum\:_{i-1}^{{N}^{b}}\frac{F\left({X}_{i}^{b}{\beta\:}^{a}\right)}{{N}^{b}}-\sum\:_{i-1}^{{N}^{b}}\frac{F\left({X}_{i}^{b}{\beta\:}^{b}\right)}{{N}^{b}}\right]$$

The explained portion of the equation comprises the initial half, whereas the unexplained portion comprises the subsequent half. In the formula, $\:{\overline{\text{Y}}}^{\text{a}}$ and $\:{\overline{\text{Y}}}^{\text{b}}$ are the mean outcomes of stunting in SC-ST and Non-SC-ST children., F is the function of the cumulative distribution of the logistic distribution, $\:{N}^{a}$ and $\:{N}^{b}$are the sample size of the two compared populations. The first term in the parentheses in the above equation represents the fraction of the inequality due to the group differences in observed characteristics and the fraction attributable to differences in estimated coefficient, and the second term represents the fraction due to differences in Y level^34–36^. Hence, in order to analyze the disparity in the prevalence of stunting between SC-ST and non-SC-ST groups, we utilized the ‘fairlie’ package in Stata-17.

## Results

### SC-ST and Non-SC-ST gap in the prevalence of stunting

Table 1 demonstrates the prevalence of stunting among SC-ST and non-SC-ST children in India. The results reveal that the overall prevalence of stunting among SC-ST children (39.60) was higher than that of non-SC-ST children (33.27%). Furthermore, the prevalence of stunting was significantly higher among SC-ST children (44.86%) compared to non-SC-ST (36.90%) aged 15–30 months. Similarly, SC-ST male children (40.52%) were more prone to stunting than non-SC-ST (33.92%). However, children from the Muslim SC-ST religion (38.95%) had a higher prevalence of stunting compared with non-SC-ST Muslim (37.06%) children.

**Table 1:** Prevalence of stunting among SC-ST and Non-SC-ST children (0–59 months age) in India.

Background Characteristics	Stunting					
	SC-ST (*N* = 83,909)			non-SC-ST (*N* = 1,09,977)		
	*n*	<-2 SD	Mean Z-score	*n*	<-2 SD	Mean Z-score
Age of child (months)						
< 15	5832	28.11	-0.91	6718	25.12	-0.73
15–30	9526	44.86	-1.65	10,738	36.90	-1.38
31–45	9135	44.78	-1.71	10,067	36.82	-1.45
46–59	8010	40.28	-1.66	8862	33.90	-1.44
		${\chi}^2$ = 1400.00	F = 845.58		${\chi}^2$ = 1200.00	F = 1047.94
		*p = < 0.05*	*p = < 0.05*		*p = < 0.05*	*p = < 0.05*
Gender of child						
Male	17,232	40.52	-1.53	19,428	33.92	-1.28
Female	15,271	38.62	-1.43	16,957	32.56	-1.22
		${\chi}^2$ = 1200.00 =72.616	t = -10.349		${\chi}^2$ = 39.931	t = -7.303
		*p = < 0.05*	*p = < 0.05*		*p = < 0.05*	*p = < 0.05*
Birth order						
1	10,460	35.44	-1.35	12,868	29.35	-1.12
2	9944	38.85	-1.46	12,372	32.59	-1.22
3+	12,099	45.47	-1.66	11,145	40.24	-1.49
		${\chi}^2$ =438.634	F = 166.24		${\chi}^2$ = 815.143	F = 334.77
		*p = < 0.05*	*p = < 0.05*		*p = < 0.05*	*p = < 0.05*
Mothers age at birth						
< 20	3965	41.81	-1.60	4264	36.77	-1.45
20–30	22,898	39.07	-1.46	27,041	33.00	-1.24
> 30	5640	40.27	-1.48	5080	31.89	-1.14
		${\chi}^2$ = 33.766	F = 28.04		${\chi}^2$ = 98.530	F = 104.43
		*p = < 0.05*	*p = < 0.05*		*p = < 0.05*	*p = < 0.05*
Mother’s delivery						
Normal	28,779	41.04	-1.53	29,605	35.39	-1.33
Caesarean	3724	32.24	-1.23	6780	26.59	-0.99
		${\chi}^2$ = 261.541	t = -15.158		${\chi}^2$ = 639.369	t = -25.286
		*p = < 0.05*	*p = < 0.05*		*p = < 0.05*	*p = < 0.05*
Mother’s BMI*						
Underweight	7824	47.07	-1.76	8000	40.89	-1.55
Normal	21,027	38.80	-1.44	22,621	33.71	-1.25
Overweight	3652	31.05	-1.24	5764	25.79	-1.00
		${\chi}^2$ =728.728	F = 350.03		${\chi}^2$ = 1200.00	F = 543.17
		*p = < 0.05*	*p = < 0.05*		*p = < 0.05*	*p = < 0.05*
Religion						
Hindu	23,905	39.89	-1.49	28,426	32.66	-1.23
Muslim	824	41.67	-1.38	7225	37.06	-1.36
Others	7774	35.89	-1.39	734	21.76	-0.83
		${\chi}^2$ = 113.77	F = 125.97		${\chi}^2$ = 250.052	F = 74.03
		*p = < 0.05*	*p = < 0.05*		*p = < 0.05*	*p = < 0.05*
Education						
No education	9861	47.41	-1.73	9112	45.78	-1.68
Primary	5661	44.06	-1.64	4658	40.08	-1.51
Secondary	15,068	36.60	-1.40	18,170	31.63	-1.22
Higher	1913	26.09	-0.97	4445	22.07	-0.78
		${\chi}^2$ = 1100.00	F = 331.34		${\chi}^2$ =2600.00	F = 804.82
		*p = < 0.05*	*p = < 0.05*		*p = < 0.05*	*p = < 0.05*
Mother’s working status						
Not working	31,243	39.54	-1.48	35,457	33.26	-1.25
Working	1260	41.33	-1.56	928	33.46	-1.30
		${\chi}^2$ =2.117	t = 2.167		${\chi}^2$ =1.101	t = 1.892
		*p* = 0.146	*p = < 0.05*		*p* = 0.294	*p* = 0.059
Wealth index						
Poorest	14,520	47.07	-1.73	8939	45.94	-1.72
Poorer	8575	40.67	-1.52	8939	39.33	-1.47
Middle	5174	36.25	-1.40	7675	33.51	-1.28
Richer	2918	30.33	-1.19	6299	27.38	-1.05
Richest	1316	25.16	-0.92	4533	22.33	-0.81
		${\chi}^2$ =1500.00	F = 311.56		${\chi}^2$ = 3000.00	F = 740.21
		*p = < 0.05*	*p = < 0.05*		*p = < 0.05*	*p = < 0.05*
Residence						
Urban	4046	33.71	-1.27	7398	28.62	-1.06
Rural	28,457	41.00	-1.53	28,987	35.23	-1.33
		${\chi}^2$ = 206.418	t = 14.028		${\chi}^2$ =404.830	t = 24.432
		*p = < 0.05*	*p = < 0.05*		*p = < 0.05*	*p = < 0.05*
Region						
North	4494	32.87	-1.30	6101	27.65	-1.05
Central	9333	45.17	-1.67	15,591	37.71	-1.40
East	5739	37.89	-1.47	4299	31.84	-1.24
Northeast	7461	35.43	-1.28	1728	32.05	-1.18
West	2682	41.93	-1.47	3660	34.12	-1.22
South	2794	34.09	-1.28	5006	27.64	-1.09
		${\chi}^2$ =691.137	F = 167.04		${\chi}^2$ =921.436	F = 171.30
		*p = < 0.05*	*p = < 0.05*		*p = < 0.05*	*p = < 0.05*
Drinking Behaviour (alcohol)						
No	31,517	39.54	-1.48	36,206	33.25	-1.25
Yes	986	44.50	-1.61	179	40.56	-1.43
		${\chi}^2$ = 0.618	t = -1.965		${\chi}^2$ =4.414	t = 1.435
		*p* = 0.432	*p = < 0.05*		*p = < 0.05*	*p* = 0.151
Smoking Behaviour						
No	28,688	39.26	-1.47	35,120	33.06	-1.24
Yes	3815	45.93	-1.74	1265	43.05	-1.64
		${\chi}^2$ = 38.427	t = 5.546		${\chi}^2$ = 61.663	t = 8.825
		*p = < 0.05*	*p = < 0.05*		*p = < 0.05*	*p = < 0.05*
Use of cooking fuel						
Clean fuel	9654	34.79	-1.32	15,473	29.00	-1.08
Polluting fuel	22,849	42.53	-1.58	20,912	38.06	-1.43
		${\chi}^2$ = 498.563	t = 22.119		${\chi}^2$ =950.042	t = 31.484
		*p = < 0.05*	*p = < 0.05*		*p = < 0.05*	*p = < 0.05*
Status of Sanitation						
Safe	18,401	35.85	-1.36	22,752	30.14	-1.14
Unsafe	14,102	44.21	-1.63	13,633	40.24	-1.50
		${\chi}^2$ = 500.939	t = 21.249		${\chi}^2$ = 954.431	t = 28.162
		*p = <0.05*	*p = < 0.05*		*p = < 0.05*	*p = < 0.05*
Status of drinking water#						
SDW	28,081	39.70	-1.49	32,982	33.44	-1.26
UDF	4422	38.79	-1.44	3403	31.45	-1.17
		${\chi}^2$ =3.881	t = -1.916		${\chi}^2$ = 8.737	t = -4.237
		*p = < 0.05*	*p* = 0.055		*p = < 0.05*	*p = < 0.05*
***Total***	32,503	39.60		36,385	33.27	

Note: BMI^*^- body mass Index; Status of drinking water ^#^; SDW-Safe drinking water, UDW-Unsafe drinking water; CI: 95%]- Confidence Interval at 95% significance level.

In terms of education and wealth status, children whose mothers had no education (47.41%) and those from the poorest wealth status (47.07%) in SC-ST groups exhibited a higher prevalence of stunting than non-SC-ST children whose mothers had lower educational attainment (45.78%) and the poorest wealth status (45.94%). Moreover, children residing in rural areas (41.00%) and those whose mothers had lower BMI (underweight) (47.07%) from SC-ST social groups were significantly more stunted compared to non-SC-ST rural residents (35.23%) and children of mothers with lower BMI (40.89%).

Additionally, mothers in SC-ST households with habits such as alcohol consumption (45.13%), smoking (46.21%), using polluting cooking fuel (42.53%), and practicing unsafe sanitation (43.70%) had children with a higher prevalence of stunting compared to non-SC-ST children of mothers who consumed alcohol (40.56%), smoked (43.05%), used polluting cooking fuel (38.06%), and practiced unsafe sanitation (30.24%. Overall, there is a 6.33 per cent gap in the prevalence of stunting between SC-ST and non-SC-ST children in India.

## SC-ST and Non-SC-ST predictors of stunting

Table 2 shows the determinants of stunting among SC-ST and non-SC-ST children 0–59 months in India. We have represented the adjusted odds ratio (AOR) of stunting for total children (combined), SC-ST, and non-SC-ST. The results revealed that children who belonged to SC-ST were 13% (AOR: 1.130, 95% CI: 1.105–1.155) more likely to be stunted than non-SC-ST children. Furthermore, the AOR from the SC-ST model revealed that children aged 15–30 months and males were 2.11 times (AOR: 2.112, 95% CI: 2.017–2.211) and 1.08 times (AOR: 1.085, 95% CI: 1.051–1.121) more likely to be stunted than their reference category respectively. However, children’s birth order and mother’s age at birth are some of the essential determinant factors significantly associated with stunting, where children whose order of birth was 3 + and mother’s age at birth was less than 20 years were 26% (AOR: 1.259, 95% CI: 1.202–1.319) and 18% (AOR: 1.182, 95% CI: 1.102–1.267) more likely to be stunted. Relatively, an inverse relationship is identified between the educational status of mothers, wealth quintile, and children stunting; as a mother’s education and wealth index decreased, the AOR of children stunting likely increased and vice-versa. Additionally, those mothers who had a lower BMI (underweight) and their children were 56% (AOR: 1.561, 95% CI: 1.473–1.654) more likely to be stunted than mothers with higher BMI (overweight). Similarly, the mothers with no education (AOR: 1.531, 95% CI: 1.422–1.647) and children belonged to the poorest wealth index (AOR: 1.821, 95% CI: 1.659–1.998) were at higher risk of stunting, compared to higher education of mothers and children from affluent wealth index household. The likelihood of stunting was also significantly higher among children who reside in the western region (AOR: 1.352, 95% CI: 1.266–1.443), mothers with smoking habits (AOR: 1.083, 95% CI: 1.006–1.166), and those households have unsafe satiation (AOR: 1.072, 95% CI: 1.033–1.114) practices compared to the reference category.

**Table 2:** Multivariable logistic regression model showing the association between stunting and socioeconomic and demographic characteristics of children aged 0–59 months in India.

Background Characteristics	Stunting [CI: 95%]	
	UOR	AOR
Social groups		
Non-SC-ST^®^	1	1
SC-ST	1.315 [1.29–1.341] ***	1.13 [1.105–1.155] ***
Age of child (months)		
< 15 months^®^	1	1
15–30 months	1.856 [1.806–1.907] ***	1.895 [1.843–1.949] ***
31–45 months	1.851 [1.801–1.903] ***	1.869 [1.817–1.923] ***
46–59 months	1.595 [1.55–1.64] ***	1.58 [1.534–1.626] ***
Gender of child		
Male	1.068 [1.049–1.089] ***	1.074 [1.053–1.095] ***
Female^®^	1	1
Birth order		
1^®^	1	1
2	1.161 [1.135–1.188] ***	1.131 [1.103–1.158] ***
3+	1.601 [1.564–1.639] ***	1.242 [1.207–1.278] ***
Mothers age at birth		
< 20	1.189 [1.146–1.234] ***	1.242 [1.19–1.297] ***
21–30	1.017 [0.989–1.045]	1.098 [1.065–1.132] ***
> 30^®^	1	1
Mother’s delivery		
Normal	1.535 [1.498–1.572] ***	1.049 [1.021–1.077] ***
Caesarean^®^	1	1
Mother’s BMI*		
Underweight	2.059 [1.996–2.124] ***	1.543 [1.492–1.595] ***
Normal	1.478 [1.44–1.517] ***	1.215 [1.181–1.249] ***
Overweight^®^	1	1
Religion		
Hindu^®^	1	1
Muslim	1.086 [1.057–1.116] ***	1.126 [1.093–1.161] ***
Others	0.824 [0.786–0.864] ***	1.001 [0.951–1.054]
Education		
No education	2.924 [2.829–3.023] ***	1.595 [1.532–1.662] ***
Primary	2.415 [2.326–2.507] ***	1.474 [1.412–1.538] ***
Secondary	1.682 [1.632–1.733] ***	1.244 [1.203–1.285] ***
Higher^®^	1	1
Mother’s working status		
Not working	0.945 [0.892–1.002] *	1.042 [0.981–1.107]
Working^®^	1	1
Wealth index		
Poorest	2.941 [2.847–3.039] ***	1.857 [1.766–1.952] ***
Poorer	2.24 [2.166–2.316] ***	1.628 [1.558–1.702] ***
Middle	1.771 [1.711–1.833] ***	1.441 [1.384-1.5] ***
Richer	1.32 [1.274–1.368] ***	1.172 [1.129–1.217] ***
Richest^®^	1	1
Residence		
Urban^®^	1	1
Rural	1.401 [1.37–1.432] ***	0.941 [0.916–0.966] ***
Region		
North^®^	1	1
Central	1.593 [1.545–1.642] ***	1.222 [1.183–1.263] ***
East	1.262 [1.217–1.309] ***	0.966 [0.929–1.005] *
Northeast	1.223 [1.148–1.304] ***	0.958 [0.894–1.026]
West	1.379 [1.328–1.432] ***	1.334 [1.282–1.388] ***
South	1.000 [0.965–1.036]	1.107 [1.065–1.151] ***
Drinking Behaviour (alcohol)		
No^®^	1	1
Yes	1.401 [1.24–1.581] ***	1.102 [0.97–1.252]
Smoking Behaviour		
No^®^	1	1
Yes	1.488 [1.413–1.567] ***	1.058 [1.001–1.118] **
Use of cooking fuel		
Clean fuel^®^	1	1
Polluting fuel	1.506 [1.478–1.535] ***	1.007 [0.981–1.034]
Status of Sanitation		
Safe^®^	1	1
Unsafe	1.547 [1.518–1.578] ***	1.097 [1.07–1.123] ***
Status of drinking water		
SDW^®^	1	1
UDW	0.948 [0.918–0.979] ***	0.908 [0.876–0.94] ***
***Log-likelihood***		-117512.11
***Pseudo r-squared***		0.047
**Chi-Square (** $\varvec{\chi}^{\textbf{2}}$ **)**		11522.226

Note: BMI^*^- body mass Index; Status of drinking water ^#^: SDW-Safe drinking water, UDW-Unsafe drinking water; AOR - adjusted odds ratio; UOR - unadjusted odds ratio; [CI: 95%]- Confidence Interval at 95% significance level; ^®^- reference category; statistical significance is denoted by asterisks where *p-value < 0.1, **p-value < 0.05, and ***p-value < 0.01.

Furthermore, the non-SC-ST model revealed the children from 15 to 30 months (AOR: 1.775, 95% CI: 1.713–1.839) and males (AOR: 1.066, 95% CI: 1.04–1.092) were more prone to stunting compared to lower age and female. However, the likelihood of stunting was higher among those children whose order of birth was 3 + and whose mother’s age at birth was less than 20 years at 22.9% (AOR: 1.229, 95% CI: 1.185–1.275) and 27.5% (AOR: 1.275, 95% CI: 1.207–1.347), respectively. Moreover, lower BMI (underweight) of mothers and Muslim religion of children were 1.5 times (AOR: 1.523, 95% CI: 1.461–1.588) and 1.13 times (AOR: 1.128, 95% CI: 1.093–1.164) more likely to be stunted compared to higher BMI and Hindu religion respectively. Similarly, mothers with no education were 66% (AOR: 1.660, 95% CI: 1.579–1.745) more likely to be stunted than the higher educational levels of mothers. Additionally, the poorest wealth index of children was 85.6% (AOR: 1.856, 95% CI: 1.747–1.972), more prone to stunting than children from the wealthiest households. The likelihood of child stunting was also higher among those who resided in the western region of the country at 31.9% (AOR: 1.319, 95% CI: 1.254–1.387) compared to the northern region. It is also found that the likelihood of stunting was significantly higher among children whose mothers had alcohol consumption habits (AOR: 1.297, 95% CI: 1.008–1.669) and unsafe satiation (AOR: 1.116, 95% CI: 1.081–1.152) practices compared to non-alcohol consumes mothers and safe satiation practices households.

## Results of the SC-STs and non-SC-STs gap decomposition

We use the Fairlie decomposition to break down the gap in stunting among two social groups (SC-ST and non-SC-ST) and quantify the contribution of different factors to explain this gap. Table 3 represents the comprehensive outcomes of the decomposition model for the differences in stunting between SC-ST and non-SC-ST children. The probability of stunting among SC-ST and non-SC-ST children was 0.395985 and 0.332671, respectively. The difference between these two social groups is 0.063315. The results revealed that 68.94% of this disparity of stunting between SC-ST and non-SC-ST children was explained by the observed variables included in the decomposition analysis. The remaining gap in stunting (31.06%), which is referred to as the “unexplained gap”, may be related to other factors that were not able to be included in the analysis. A graphical representation of the distribution of each contributor is presented in **Supplementary Fig. 1.**

**Table 3:** Fairlie decomposition of disparity on stunting between SC-ST and non-SC-ST children in India.

Terms of decomposition					Stunting
Mean Prediction among SC-ST Children					0.395985
Mean Prediction among Non-SC-ST Children					0.332671
Difference					0.063315
Total explained					0.043649
Explained (%)					68.94
Unexplained (%)					31.06
Variables Contribution to Difference	Coefficient	p-value	[CI: 95%]		Contribution (%)
			Lower	Upper	
Age of child (months)	-0.001311	0.000	-0.001450	-0.001172	-2.070
Gender of child	-0.000205	0.000	-0.000282	-0.000129	-0.324
Birth order	0.002103	0.000	0.001751	0.002456	3.322
Mothers age at birth	0.000702	0.000	0.000454	0.000950	1.109
Mother’s delivery	0.000939	0.025	0.000118	0.001760	1.483
Mother’s BMI	0.006975	0.000	0.006182	0.007768	11.017
Religion	0.000184	0.317	-0.000177	0.000545	0.291
Education	0.008143	0.000	0.006780	0.009506	12.861
Mother’s working status	-0.000079	0.353	-0.000247	0.000088	-0.125
Wealth index	0.026130	0.000	0.022977	0.029283	41.270
Residence	-0.000545	0.285	-0.001546	0.000455	-0.861
Geographical region	-0.000120	0.100	-0.000263	0.000023	-0.190
Alcohol	-0.000180	0.277	-0.000504	0.000145	-0.284
Smoking Behaviour	0.000260	0.324	-0.000257	0.000778	0.411
Use of cooking fuel	-0.001671	0.019	-0.003069	-0.000273	-2.639
Status of Sanitation	0.002694	0.000	0.001519	0.003869	4.255
Status of drinking water	-0.000381	0.000	-0.000594	-0.000167	-0.601

The findings indicated that economic status (wealth index) was the main contributor, explaining about 41.27% of the disparity in stunting between SC-ST and non-SC-ST children. Mothers’ education and mothers’ BMI were other essential contributors explaining 12.86% and 11.02% of the gap in children stunting between SC-ST and Non-SC-ST groups, respectively. The status of sanitation facilities was also explained by 4.26% of the gap in stunting. The results also explained that the children’s birth order (23.32%) and mother’s type of delivery (1.48%) also significantly contributed to the difference in the stunting between SC-ST and non-SC-ST children. Conversely, the factors, including use of cooking fuel (-2.64), age (-2.07%), drinking water (-0.60%), and gender of the children (-0.324%) have a negative contribution, which significantly indicates the reduction of the stunting gap between the SC-ST and non-SC-ST children. However, religion and smoking behaviour had positively contributed to inequality, and working status, geographical region, alcohol consumption, and residence are the negative contributors to stunting inequality but became statistically insignificant in the decomposition analysis.

## Discussion

The study aimed to measure and analyse the socioeconomic inequality in stunting among SC-ST and non-SC-ST children in India. The present study shows a significant outcome in that a substantial gap in stunting rates was observed across two social groups. Furthermore, the findings revealed that between SC-ST (39.60%) and non-SC-ST (33.27%) social groups of children, there was a significant difference of 6.33 per cent in stunting.

The results revealed that children (combined) from the poorest households were 85.7 per cent more likely (AOR: 1.857, 95% CI: 1.766–1.952) to be stunted than those from affluent households. This indicated that the lower wealth status of the children determined a higher stunting probability. Moreover, SC-ST children were more vulnerable to stunting compared to non-SC-ST groups. Previous literature found that SC-ST children were inferiorly malnourished with differences in lower economic conditions, which is a strong predictor of childhood stunting^37^. Consisting with this, the wealth index contributed 41.27% in stunting inequality between SC-ST and non-SC-ST in India. This suggests wealth is the crucial determinant for the differences in stunting of deprived castes/ groups in society from certain mainstream societal groups in India^38^. Evidence suggests that household income level influences the average per capita consumption of calories, protein, and fat; economically privileged households had higher levels than those in poverty^39^. Moreover, economic differences potentially accelerated stunting development among children in the poorest economic (wealth quintile) condition^11,40^.

Likewise, maternal education was strongly correlated with children stunting, accounting for 12.86% of the inequality in stunting between SC-ST and non-SC-ST. This implies that higher-educated mothers may anticipate the financial resources more wisely and improve their children’s nutritional well-being^41^. A well-educated mother would know the essential nutritional knowledge and be cognizant of the adverse consequences of a child’s health^28,40^. Furthermore, the mother’s nutritional status (BMI) is significantly correlated with stunting of the children, which is consistent with global studies^23^. In terms of group inequality, SC-ST women who were underweight (47.07%) had a higher prevalence of child stunting than non-SC-ST underweight mothers (40.89%). Initially, the mother’s BMI contributed 11.02% to the stunting difference between SC-ST and non-SC-ST children in India. Prior investigations have established a robust correlation between the mother’s nutritional health and their children’s nutritional status, with child stunting being more common among underweight mothers^42,43^. Similarly, earlier evidence revealed that children born to underweight mothers are more likely to be stunted due to low birth weight^42,44^.

Sanitation practices are yet another significant contributing factor to stunting inequality by 4.26% among SC-ST and non-SC-ST children in the country. Moreover, unsafe or unhygienic sanitation practices were imposed on SC-ST groups due to caste-based prejudice and a lack of inclusive and sustainable policy implementation^33^. Furthermore, global evidence illustrated a significant association between sanitation facilities and stunting exists, characterizing inadequate poor sanitation practices as a constraint to linear growth in children^29,33,45–48^. The reasons for explaining this are that children began crawling, walking, and placing objects in their mouths, and they became more sensitive to environmental contamination, potentially increasing the risk of bacterial diseases. This causes a nutritional decrease in youngsters, resulting in undernutrition^33^. Previous literature suggested that hygienic sanitation causes stunting in even well-fed children, hence children without safe hygiene do not grow well^29^. This could imply that improved sanitation practices along with balanced nutrition can reduce childhood stunting in India across nations^48,49^.

The study demonstrated a significant association between children’s mode of delivery and stunting, with conventional delivery being more strongly correlated than cesarean delivery. In regards to decomposition results, it revealed that a method of mother’s delivery contributes 1.48% to the stunting gap between SC-ST and non-SC-ST children in India. It was hypothesized that children born into SC-ST families would mostly be delivered by normal or conventional methods, while the majority of non-SC-ST families preferred a cesarean section. This phenomenon arises when doctors prefer cesarean deliveries due to their expedience and convenience, hence incentivizing wealthy and privileged families to choose this approach, potentially resulting in stunting^21,50,51^. Our study stands that cesarean birth is inversely associated with childhood stunting and has a lower stunting rate, which aligns with another finding^52^. On the contrary, children delivered by non-caesarean section were considerably less likely to be stunted than children delivered via caesarean Sects^20,21^. In the part of the inequality of stunting caused by differences in the effects of the birth order, it is one of the significant factors contributing by 3.32% among SC-ST and non-SC-ST. We found that the differences in the coefficients of having a lower birth order were associated with lower occurrences of stunting, which was recognized by a study in India^37^. Moreover, evidence shows that children with third-order births were significantly more likely to be stunted than children with first-order births^52^. One possible reason for this correlation is that household has more children, and there is a corresponding depletion of food and other vital resources, rendering it unfeasible to provide adequate sustenance and healthcare.

This study rigorously identified the inequality and contributing factors in stunting across two social groups (SC-ST and non-SC-ST) in India. It revealed that SC-ST children were more prone to stunting across different socioeconomic and demographic backgrounds. Children from the SC-ST group have more vulnerability concerning the probability of being stunted due to poor maternal education and nutritional status, lower economic conditions, and unsafe sanitation practices in households. Therefore, improving economic position, maternal education, mother’s nutritional status (BMI), and safe sanitation practices can reduce childhood stunting and need to be taken into consideration^45^.

However, to accelerate the progress on child stunting in India extensively, efforts such as the Integrated Child Development Scheme, Poshan Abhiyan, National Health Mission, Mid-Day Meal Scheme, and Targeted Public Distribution System have successfully executed a multifaceted strategy^53^. Despite the efforts across the country, the burden of child stunting still persists in India, especially when it is higher among the marginalized social group (SC-ST). A common feature across the study was the urge to implement community-based programmes and interventions that complemented national-level efforts across women’s educational attainment, nutritional outcome, household wealth status, and sanitation programs. Hence, considering the present study, policymakers should focus on improving the contributing factors of inequality, such as the utilization of public policies on children’s nutrition, women’s economic empowerment, maternal education and nutrition, mother-child delivery care, and primary health care welfare. The policy design needs to expand beyond narrower, focusing on economic and healthcare enhancement to achieve Sustainable Development Goal target 2.2 (SDG-2.2), which ensures the end of all forms of malnutrition, including stunting in children under five years of age, by 2025 ^55,56^.

Moreover, the pathways by which pathogen exposure and ingestion lead to environmental enteric dysfunction and immune impairment start with insufficient sanitation provision^56^. This allows detrimental hygiene practices such as feeding a kid without handwashing, discarding child faces in open waste piles and inadequate food storage, which are more common in disadvantaged groups^56,57^. Neglecting the environmental drivers of child stunting significantly constrains the efficacy of critical nutrition-specific interventions. Further, expedited progress in child stunting necessitates a significant transformation in both policy formulation and execution. Social benefits transfers must be strengthened for the backward social groups to enable the poorest households to improve food security and adequacy of diet. Children’s stunning cannot be reduced sustainably across social groups without addressing the nutritional, educational attainment and economic scenario. Furthermore, our findings underscore the need to focus on SC-ST groups and poor levels of education and nutrition to reduce this burden. Therefore, it is essential to conduct further research and re-evaluate existing policies to understand the reasons behind this.

This study has some crucial limitations that are acknowledged. First, this study was based on cross-sectional design data, which cannot establish a definitive cause-and-effect inference. Second, the study is limited to children aged 0–59 months only. Third, the data we used was the self-reported response by the mothers recorded in NFHS-5, which may have been influenced by bias. Fourth, the entire study and the results were computed across SC-ST and non-SC-ST categories, which may differ from other social group inequality studies.

## Conclusion

Our study explicitly illustrated a higher stunting rate among children from the SC-ST social group. Furthermore, a significant social group difference exists in stunting across SC-ST and non-SC-ST children aged 0–59 months in India. This study indicated that household wealth status, women’s educational attainment, mother’s nutritional status (BMI), sanitation status, mode of delivery, and birth order of the children contributed to this gap. The urgent urges will be expanding the quality and availability of health education and social benefits to improve health outcomes. Therefore, this study strongly supports the need for enhancing maternal education and women’s empowerment to promote their health and capacity to care for their children; implementing social protection programmes to augment purchasing power and access to services and amenities; and ensuring improved and safeguarding hygiene, sanitation, and water quality, and awareness of children’s nutrition are recommended to reduce this inequality. The operational mandate should be firm, including strengthened existing policies, intervention for deprived social groups, and a monitoring-evaluation system to achieve SDG Target 2.2.

## Electronic supplementary material

Below is the link to the electronic supplementary material.

Supplementary Material 1

## Data Availability

The current study used a dataset from the National Family Health Survey round five (NFHS-5), which has open access to all and is freely available in the Demographic and Health Surveys (DHS) repository at https://dhsprogram.com. The public can easily access this data by registering and sending online requests to the portal for significant research purposes.
