# Supplementary material for: Decomposing social groups differential in stunting among children under five in India using nationally representative sample data
Source: Sci Rep. 2024 Nov 8;14:27260. doi: 10.1038/s41598-024-78796-3 (PMC11549304; doi:10.1038/s41598-024-78796-3)
Supplement: Supplementary file 1 — Supplementary Material 1 [file 41598_2024_78796_MOESM1_ESM.docx]

Supplementary Fig 1

**Supplementary Fig 1:** Percentage contribution of the covariables to the inequality in stunting between SC-ST and non-SC-ST children (Fairlie decomposition).
